# Pneumatosis Cystoides Intestinalis: A Rare Benign Cause of Pneumoperitoneum

**DOI:** 10.1155/2013/353245

**Published:** 2013-08-01

**Authors:** Puneet Devgun, Hal Hassan

**Affiliations:** Department of Radiology, H066, Penn State Milton S. Hershey Medical Center, 500 University Drive, P.O. Box 850, Hershey, PA 17033, USA

## Abstract

Pneumatosis cystoides intestinalis is a rare gastrointestinal complication in the course of connective tissue diseases, especially in scleroderma, that can lead to pneumoperitoneum or obstruction. Findings on plain radiography may reveal radiolucent linear or bubbly circular air bubbles in the bowel wall, with or without free gas accumulation in the peritoneal cavity. Treatment of pneumatosis cystoides intestinalis ranges from supportive care to laparotomy.

## 1. Background

Pneumatosis cystoides intestinalis (PCI) is a rare gastrointestinal complication in the course of connective tissue diseases (CTD), especially in scleroderma. Pneumatosis cystoides intestinalis was first described by Du Vernoi in a cadaveric dissection in 1783. This complication is characterized by the presence of multiple gas-filled cysts in the submucosa and/or subserosa wall of the intestine [1, 2]. Although this condition is usually asymptomatic and incidentally found during radiological evaluation or laparotomy, it can be a cause of pneumoperitoneum or obstruction. Clinical signs and imaging features in these situations may mimic true abdominal viscera perforation, so a correct diagnosis is imperative as treatment of PCI is generally conservative.

Here we describe a case of pneumatosis cystoides intestinalis in the setting of scleroderma.

## 2. Presentation

**Figure 1:**
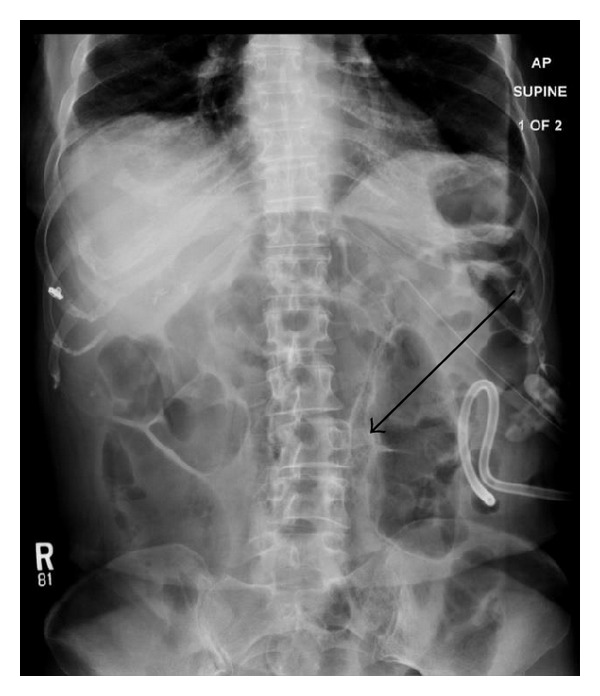
Abdominal radiograph demonstrates gastrostomy tube and jejunostomy tube in the left upper quadrant of the abdomen. Multiple small round lucencies are seen in the wall of loops of small bowel in the midabdomen (arrows).

**Figure 2:**
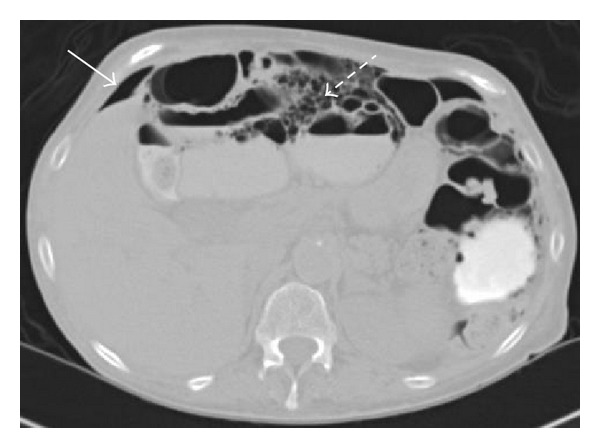
Axial CT image with enteric contrast demonstrates pneumoperitoneum in the right upper quadrant of the abdomen, anterior to the liver (arrow). Multiple large “cysts” are seen within the wall of the small bowel (dashed arrow), consistent with “pneumatosis cystoides intestinalis” seen in this patient with scleroderma. Contrast is noted in a loop of small bowel at the left side, without extravasation of contrast from the small bowel.

**Figure 3:**
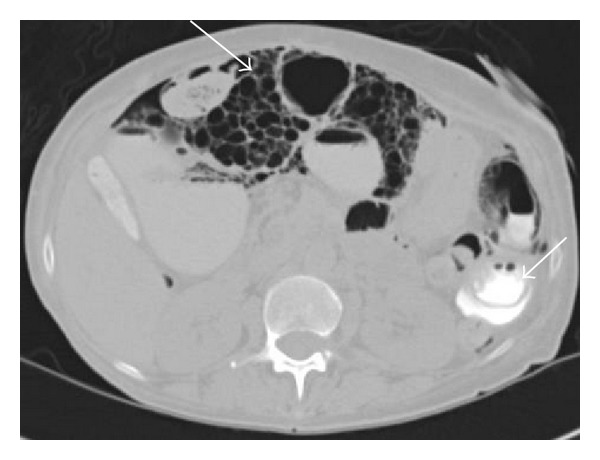
Axial CT image with enteric contrast demonstrates multiple large “cysts” within the wall of the small bowel (arrows). Enteric contrast is visualized within the lumen of small bowel at the left side of the abdomen, without extravasation (dashed arrows).

**Figure 4:**
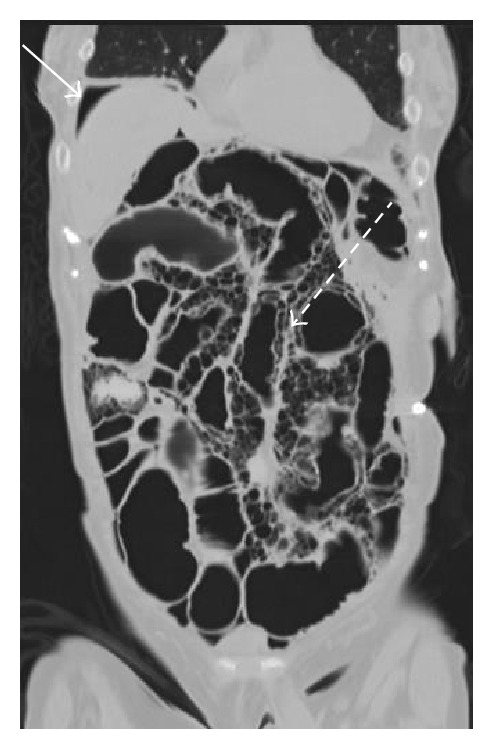
Coronal CT image demonstrates pnemo periotneum in the right upper quadrant of the abdomen, above the liver (arrow). Multiple large “cysts” are seen within multiple loops of small bowel (dashed arrows), consistent with “pneumatosis cystoides intestinalis” seen in this patient with scleroderma.

A 65-year-old female presented with leaking fluid and pain around her jejunostomy (J) tube. She had a long history of scleroderma requiring J tube placement for supplemental feeding. On examination, there were pain and tenderness around her J tube. Initial X-rays were done to assure proper location of her J tube and demonstrated contrast contained within loops of bowel; however, the bowel wall had a “mottled” appearance, and incidental note was made of pneumoperitoneum (Figure 1). Follow-up fluoroscopic J tube study the next day revealed irregular filling of the small bowel, suspicious for contrast extravasation outside of the small bowel lumen. Further confirmation by CT was performed which revealed endoluminal termination of jejunostomy catheter with no dissection of contrast into an intramural location, and note was made of worsening small bowel pneumatosis and pneumoperitoneum (Figures 2, 3 and 4). The patient was completely asymptomatic, treated conservatively with bowel rest and maintenance of fluids, and subsequently discharged home without surgical intervention.

The patient's past medical history consisted of presenting to another hospital with acute abdominal discomfort. She had a CT scan done that showed air in the peritoneal cavity suggesting a perforated bowel. She went for an exploratory laparotomy which showed no evidence of bowel rupture. A second exploratory laparotomy was done to ensure a correct diagnosis and also found no evidence of bowel rupture. The patient has a history of a severe form of scleroderma affecting the bowel with thinning of the wall and air dissection into the bowel wall (pneumatosis cystoides intestinalis) and with resultant air leaking into the peritoneal cavity. PCI is a consequence of bowel involvement in patients with scleroderma and can cause pneumoperitoneum with bowel cyst rupturing into the peritoneal cavity, mimicking bowel perforation.

## 3. Discussion

Pneumatosis cystoides intestinalis (PCI) is a rare condition characterized by the presence of air-filled cysts present in the bowel wall and mesentery and may occur anywhere in the gastrointestinal tract. The cysts (0.5–10 cm in size) are found most frequently in the terminal ileum and rarely in the proximal small bowel, stomach, and colon [2]. It is a benign condition that often responds to conservative management, but it may be a harbinger of end-stage disease, particularly in progressive systemic sclerosis [3, 4]. When the air-filled cysts rupture, they cause a pneumoperitoneum, which often is benign in nature.

Intestinal involvement in progressive systemic sclerosis, dermatomyositis (DM) or polymyositis (PM), and mixed CTD may be complicated by PCI. The pathogenesis of PCI is not fully understood, but several mechanisms have been suggested. Mucosal break down from steroids and other immunosuppressive agents cause Peyer's patches in the bowel wall to shrink, leading to an alteration of mucosal integrity and the potential for air dissection. These immunosuppressive agents also impair tissue repair mechanisms, further exacerbating ulceration and bowel necrosis. The bacterial theory suggests that bacterial fermentation of carbohydrates within the gastrointestinal tract leads to excessive gas formation, inducing absorption of this gas into the bowel wall [5]. The bacterial theory is also supported by the observation that benign PCI often responds to dietary changes, antibiotic therapy, and oxygen, which is toxic to an aerobic intestinal flora and may create a diffusion gradient across cyst wall, accelerating cyst resolution [6, 7]. In systemic sclerosis and related conditions, combined pathology such as microangiopathy, intestinal atrophy and fibrosis, impaired intestinal motility, bacterial overgrowth, elevated intraluminal pressure, and increased permeability of intestinal wall may predispose to PCI [8]. 

Gastrointestinal involvement in scleroderma is characterized by atrophy of the muscularis propria and its replacement by collagen tissue. Manometric and electrophysiological studies have shown evidence of a neuropathy of the enteric nervous system in the early stages of the disease, resulting in disturbances of digestive peristalsis and leading to gastroparesis, bacterial overgrowth of the small intestine, or constipation. Although any part of the gastrointestinal tract may be involved, the organs involved in decreasing order of frequency are the esophagus, small bowel, colon, and stomach [5]. 

Suggestive findings on plain radiography comprise different pattern of radiolucent linear or bubbly circular air bubbles in the bowel wall, with or without free gas accumulation in the peritoneal cavity. CT imaging is the gold standard procedure for the diagnosis of PCI. Furthermore, CT allows the detection of additional findings that may suggest an underlying potentially worrisome cause of PCI such as bowel wall thickening, altered contrast mucosal enhancement, dilated bowel, soft tissue stranding, ascites, and the presence of portal air.

Treatment of pneumatosis cystoides intestinalis ranges from supportive care to laparotomy. PCI is often benign and only conservative treatment, and followup is warranted. Surgery is generally indicated in patients with severe pain. The decision to proceed with explorative laparotomy must be based on the thorough analysis of a detailed history, physical examination, laboratory tests, and radiological studies.
